# Association between ocular biometric parameters and scleral rigidity as measured by fundus pulsation optical coherence elastography in eyes with and without glaucoma

**DOI:** 10.3389/fmed.2026.1835538

**Published:** 2026-05-19

**Authors:** Karine D. Bojikian, Zhaoyu Gong, Morgan Hansen-Oja, Sarah Lee, Andrew Chen, Eric Duerr, Raghu C. Mudumbai, Ruikang K. Wang, Philip P. Chen

**Affiliations:** 1 Department of Ophthalmology, University of Washington, Seattle, WA, United States; 2 Department of Bioengineering, University of Washington, Seattle, WA, United States

**Keywords:** axial length, corneal hysteresis, corneal pachimetry, fundus pulsation, glaucoma

## Abstract

**Purpose:**

To evaluate the correlation between ocular rigidity, as measured using fundus pulsation optical coherence elastography (FP-OCE), and ocular and systemic biometric parameters in patients with and without glaucoma.

**Methods:**

Primary open-angle glaucoma (POAG) and normal eyes were scanned using swept-source OCT (PLEX Elite 9000, Carl Zeiss Meditec). For each scan, a 3-mm field centered at the fovea was continuously monitored for 3000 B-scans, which were then processed to extract the ratio of maximal choroidal deformation to choroidal thickness [choroidal maximal strain (CMS)]; ocular rigidity is presumed to be the inverse of CMS. CMS was analyzed for association with pertinent ocular and systemic parameters.

**Results:**

Primary open-angle glaucoma eyes (*N* = 58) had lower corneal hysteresis (CH) compared to normal eyes (*N* = 36) (*p* < 0.001), but no other significant differences in demographics and ocular parameters were found. CMS was significantly correlated with increasing axial length (AL) in POAG and normal eyes (*p* ≤ 0.033); with increasing central corneal thickness (CCT) and CH in normal (*p* ≤ 0.042), but not in POAG eyes; and with increasing age in POAG (*P* = 0.049), but not in normal eyes. CMS was not associated with intraocular pressure, blood pulse amplitude, or ocular perfusion pressure.

**Conclusion:**

This study found that ocular rigidity, as indirectly measured by FP-OCE of the posterior sclera, is inversely associated with AL in both normal eyes and eyes with glaucoma, with CH and CCT in normal eyes, and with age in POAG eyes. Nevertheless, establishing a causal link with glaucoma development and progression will require prospective longitudinal investigation.

## Introduction

Glaucoma is one of the leading causes of irreversible blindness worldwide, affecting an estimated 76 million individuals, with projections indicating a steady rise in prevalence due to the aging global population (1). The disease is multifactorial in nature, with numerous risk factors contributing to its onset and progression. Elevated intraocular pressure (IOP) remains the most important modifiable risk factor; however, the exact mechanism by which elevated IOP leads to glaucomatous optic neuropathy remains unclear. One hypothesis suggests that reduced scleral stiffness (i.e., decreased rigidity) may result in greater deformation (strain) of the lamina cribrosa (LC), increasing the risk of axonal damage under elevated IOP (2). Other well established risk factors for glaucoma diagnosis and progression include thin central corneal thickness (CCT) (3) and lower corneal hysteresis (CH) (4–6), indicating that corneal biomechanics may play a direct role or serve as a biomarker for other contributors to glaucoma pathophysiology, as well as longer axial length (AL), possibly due to thinner LC (7, 8), which may contribute to a heightened susceptibility to glaucomatous damage.

Historically, the assessment of scleral rigidity has been performed using several invasive and time-intensive techniques, some of which include anterior chamber manometry (9), Schiotz tonometry (10), and biaxial mechanical testing (11). While these methods have provided valuable insights into ocular biomechanics, their practical application in routine clinical settings has been limited due to their complexity and invasiveness. Fundus pulsation optical coherence elastography (FP-OCE) is a promising alternative to assess ocular rigidity *in vivo*. It is based on a choroidal pulsation model, where the choroidal volume change is facilitated by blood vessel flux with each cardiac cycle but restrained by posterior sclera. The ocular rigidity measured on the posterior sclera is thus inversely proportional to choroidal maximal (max) strain (CMS), which can be obtained through phase-sensitive optical coherence tomography (PhS-OCT) measurement of choroidal deformation and thickness (12). The clinical validation of FP-OCE on a myopia cohort provided strong evidence to the validity and repeatability of this methodology (12).

This study aims to evaluate the relationship between *in vivo* ocular rigidity, as measured by CMS, and pertinent systemic and ocular biometric parameters in individuals with and without glaucoma, with the goal of gaining a deeper understanding of ocular biomechanical properties and their implications in glaucoma pathophysiology.

## Materials and methods

### Subjects

The study was approved by the institutional review board of the University of Washington (UW), and informed consent was obtained from all subjects before imaging. This study followed the tenets of the Declaration of Helsinki and was conducted in compliance with the Health Insurance Portability and Accountability Act.

Subjects with diagnosis of primary open angle glaucoma (POAG) or with normal optic disks were prospectively enrolled at the UW Eye Institute. All subjects underwent a comprehensive ophthalmologic examination at time of enrollment followed by CCT measurements (PachPen, Accutome Inc., Malvern, PA, USA), CH measurements (Ocular Response Analyzer, ORA, Reichert, Inc.), AL measurements through ophthalmic biometry obtained with partial coherence interferometry (IOL Master 500, Carl Zeiss Meditec Inc., Dublin CA, US), optical coherence tomography (OCT) imaging (PLEX Elite 9000, Carl Zeiss Meditec Inc., Dublin CA, US) and blood pressure (BP) measurements (Welch Allyn Model LXI #4700-60; Welch Allyn, Skaneateles Falls, New York) in a seated position to calculate blood pulse amplitude (BPA) and mean ocular perfusion pressure (MOPP). MOPP was defined as 2/3 (mean arterial pressure) - IOP, where mean arterial pressure = diastolic BP + 1/3(systolic BP − diastolic BP). Glaucoma subjects also received a visual field (VF) test to determine mean deviation (MD) and pattern standard deviation (PSD) and were divided into 3 severity stages (mild, moderate, and severe) based on the VF MD value: mild stage = MD better than −6.00 dB; moderate stage = MD −6.00 dB to −12.00 dB; severe stage = MD worse than −12.00 dB. All VF tests were performed on Humphrey Field Analyzer II (Carl Zeiss Meditec, Dublin, CA).

The diagnosis of POAG was based on a documented IOP > 21 mmHg, the presence of characteristic glaucomatous optic neuropathy and an abnormal RNFL thickness on spectral-domain (SD)–OCT irrespective of VF loss. Exclusion criteria included any significant media opacity preventing high-quality imaging, an AL greater than 28 mm, and any previous intraocular surgeries other than uncomplicated glaucoma or cataract surgery. If both eyes met the eligibility criteria, both were included.

### Image acquisition and scanning protocol

Fundus pulsation optical coherence elastography examination was randomly conducted by three certified operators (ZG, MH, SL) on a commercial swept-source OCT (PLEX Elite 9000, Carl Zeiss Meditec) with a customized B-Scan scanning protocol to accommodate the FP-OCE examination. The OCT device has a central wavelength of 1060 nm, with an optical bandwidth of 100 nm, providing an axial resolution of 6.3 μm in tissue. The lateral resolution on the fundus (estimated based on the beam size at the pupil) is 20 μm.

The customized B-Scan protocol was configured to acquire repeated B-scans over time. The dataset from every individual scan consists of an ensemble of 3000 B-scans that was repeatedly collected at one consistent cross-section centered at the fovea. Each B-scan contains 300 A-scans, spanning a 3-mm line field, oriented nasal-temporally. An A-scan contains 1536 pixels, covering a depth range of 3 mm. During the acquisition, a built-in eye-tracking module was responsible for detecting and compensating for eye movement. Large eye movements can significantly lengthen the acquisition time due to the necessary interruption for the tracking module to re-center the scanning field. Depending on the occurrence of the eye movement, the typical observed scan time is approximately 14–30 s.

### Data processing

**FIGURE 1:**
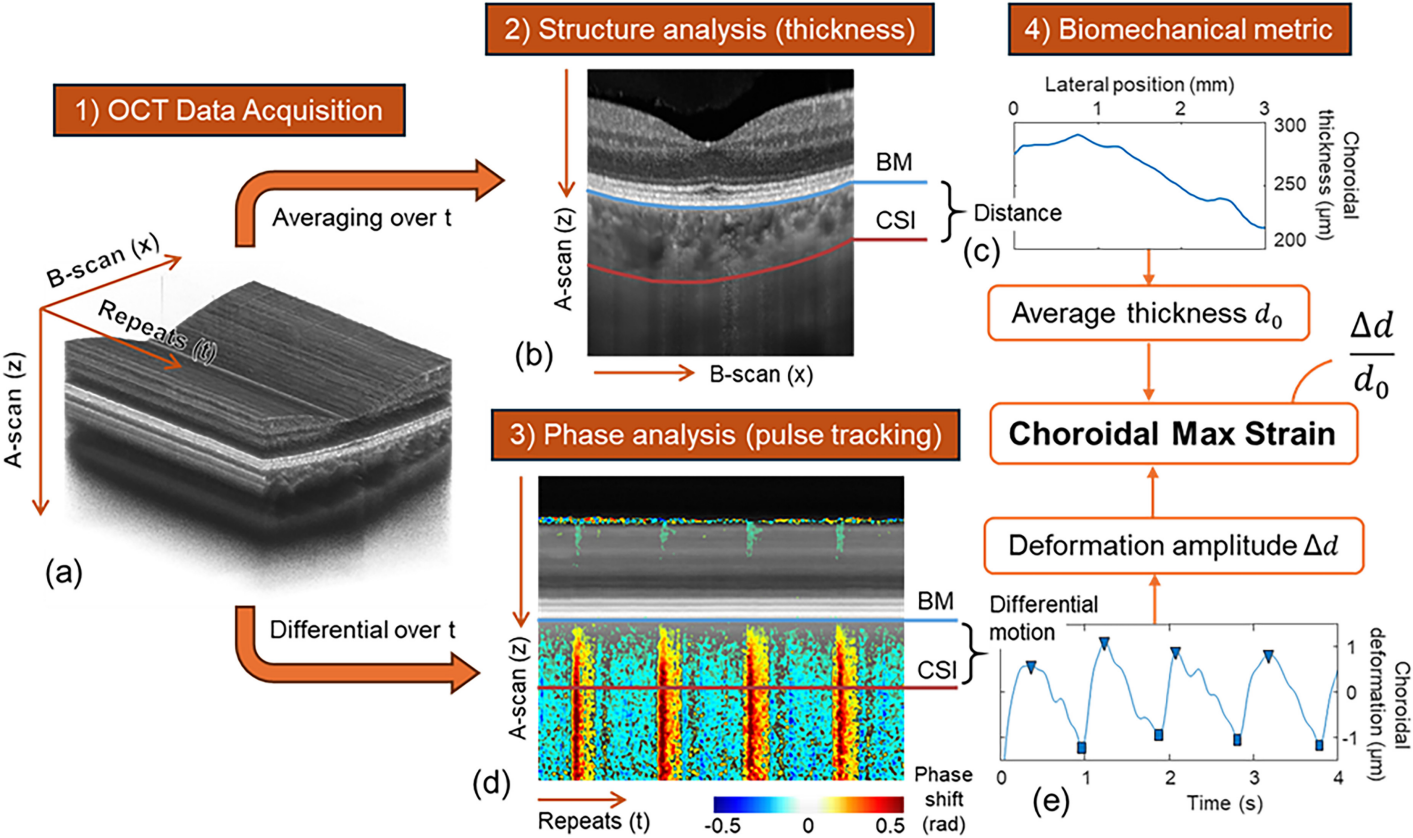
The data processing framework. **(a)** 3-D rendering of the repeated B-scan movie. **(b)** OCT structural image. **(c)** Choroidal thickness as a function of lateral position. **(d)** OCT phase shifts indicating the fundus pulsation. **(e)** Pulsatile deformation of the choroidal slab.

The FP-OCE data was processed on MATLAB 2023b (MathWorks, Inc., USA). Detailed processing steps can be found in our previous publication (12). In brief, CMS, defined as the ratio between choroidal maximal deformation and choroidal thickness, was extracted through the following steps: (1) Preprocessing (Figure 1a): from the 3000 B-scans, the ensemble of 1500 continuous B-scans that contains the least tracking-induced interruption was identified from each scan volume by automatically searching through the B-scan timestamp. Bulk motion between adjacent B-scans was minimized using a sub-pixel registration algorithm (13) and a phase compensation algorithm (14). (2) Structural analysis (Figures 1b, c): OCT structural image (Figure 1b) was obtained by averaging the intensity of all B-scans over the time axis; the choroidal thickness (*d*_0_) was measured as the average distance between Bruch’s membrane (BM) and choroid-sclera interface (CSI), see Figure 1c. (3) Phase analysis (Figures 1d, e): OCT phase shifts were obtained by differentiating the phase of every two adjacent B-scans (Figure 1d). This phase shift is fundamentally proportional to the instantaneous axial displacement (along *z*-axis) of the tissue (15). Therefore, the differential motion between BM and CSI indicates the pulsatile deformation of the choroidal slab, see Figure 1e. This pulsatile deformation was tracked throughout the time to find its amplitude (Δ*d*). (4) Choroidal max strain (CMS) was calculated from the ratio of the choroidal maximal deformation Δ*d* from step 3 and the choroidal thickness *d*_0_ from step 2. CMS is a dimensionless value, analogous to the percentage, representing how much the choroid expands in thickness. CMS equals zero means that the choroid has no thickness change during cardiac cycles, while 1 means the choroid thickness increases by 100% during cardiac cycles.

### Statistical analysis

All statistical analysis was performed with MATLAB 2023b (MathWorks, Inc., USA). Independent sample 2-tail *t*-tests based on a linear mixed-effects (LME) model were used to detect if there were any differences in age and ocular parameters among normal and POAG eyes. A LME regression was employed to evaluate the relationship between choroidal max strain and ocular parameters. The LME model was implemented by a MATLAB built-in function fitlme(). Whereas an ordinary linear regression estimates only a population level intercept and slope (the fixed effects), the LME model further incorporates patient specific random effects to account for potential intra-patient correlation in cases where both eyes were included (16). This random effect was modeled as a subject-specific random intercept. The statistical significance was set at *p* < 0.05 for all analyses.

For analyses that reached statistical significance, we additionally assessed statistical power to determine whether the available sample size was sufficient to support a well-powered analysis. The power analysis is conducted using a Monte Carlo simulation method (17).

## Results

**FIGURE 2:**
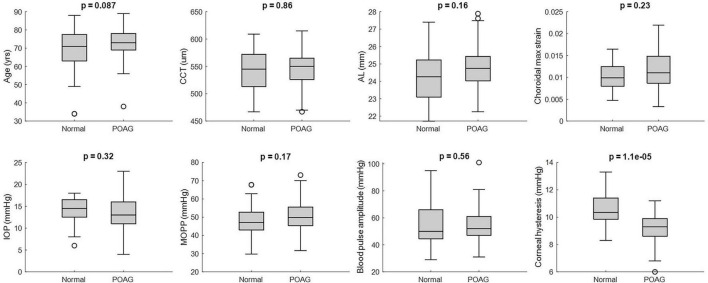
Linear mixed effects of patient’s age, axial length (AL), corneal hysteresis (CH) and central corneal thickness (CCT) for normal (*N* = 36) and primary open angle glaucoma (POAG) (*N* = 58) eyes.

Ninety-four eyes from 70 patients, including 58 POAG and 36 normal eyes, were included in the final analysis. The mean age for normal and POAG patients were 68.9 ± 12.6 and 72.9 ± 8.1 years, respectively (Table 1). In the POAG group, the average VF MD was −3.93 ± 5.65 dB. Linear mixed effects *t*-test showed no significant differences in age, IOP, MOPP, BPA, CCT, AL, or CMS were found between POAG and normal eyes (*p* ≥ 0.087) (Figure 2). However, CH was found to be significantly lower in POAG eyes compared to normal eyes (*p* < 0.001). Additionally, eyes defined as mild glaucoma by VF MD had higher CMS when compared to normal eyes (0.0126 vs. 0.0103; *p* = 0.042) and when compared to severe glaucoma eyes (0.0126 vs. 0.0081; *p* = 0.003).

**TABLE 1:** Baseline demographic information and systemic and ocular biometric parameters for normal and primary open angle glaucoma (POAG) groups.

Demographic information and systemic and ocular biometric parameters	Normal (*N* = 36)	POAG (*N* = 58)	*P*-value
Age (yrs)	68.9 ± 12.6	72.9 ± 8.1	0.087*
Male	15 (41.7%)	27 (46.6%)	0.634¥
Female	21 (58.3%)	31 (53.4%)	
IOP (mmHg)	14.1 ± 3.2	13.2 ± 3.5	0.322*
Mean ocular perfusion pressure (mmHg)	48.1 ± 8.3	50.3 ± 8.7	0.174*
Blood pulse amplitude (mmHg)	54.3 ± 14.9	54.6 ± 11.9	0.564*
Number IOP lowering meds	N/A	1.6 ± 1.2	
Number of eyes on topical prostaglandin analogs	N/A	43 (74.1%)	
Visual field mean deviation (dB)	N/A	−3.93 ± 5.65	
Glaucoma severity			
Mild (MD > −6 dB)	N/A	43 (74.1%)	
Moderate (MD −6 to −12 dB)		6 (10.3%)	
Severe (MD < −12 dB		9 (15.6%)	
History of glaucoma surgery (yes)	N/A	12 (20.7%)	
Central corneal thickness (uM)	542.5 ± 38.8	543.1 ± 33.7	0.862*
Corneal hysteresis (mmHg)	10.6 ± 1.2	9.2 ± 1.1	**<0.001** *
Axial length (mm)	24.3 ± 1.3	24.8 ± 1.3	0.160*
Choroidal maximal strain	0.0103 ± 0.003	0.0117 ± 0.0045	0.237*

*2 sample *t*-test; ¥, Pearson chi-square; IOP, intraocular pressure. All values are percentages or mean ± standard deviation. Bold indicates statistically significant value.

**FIGURE 3:**
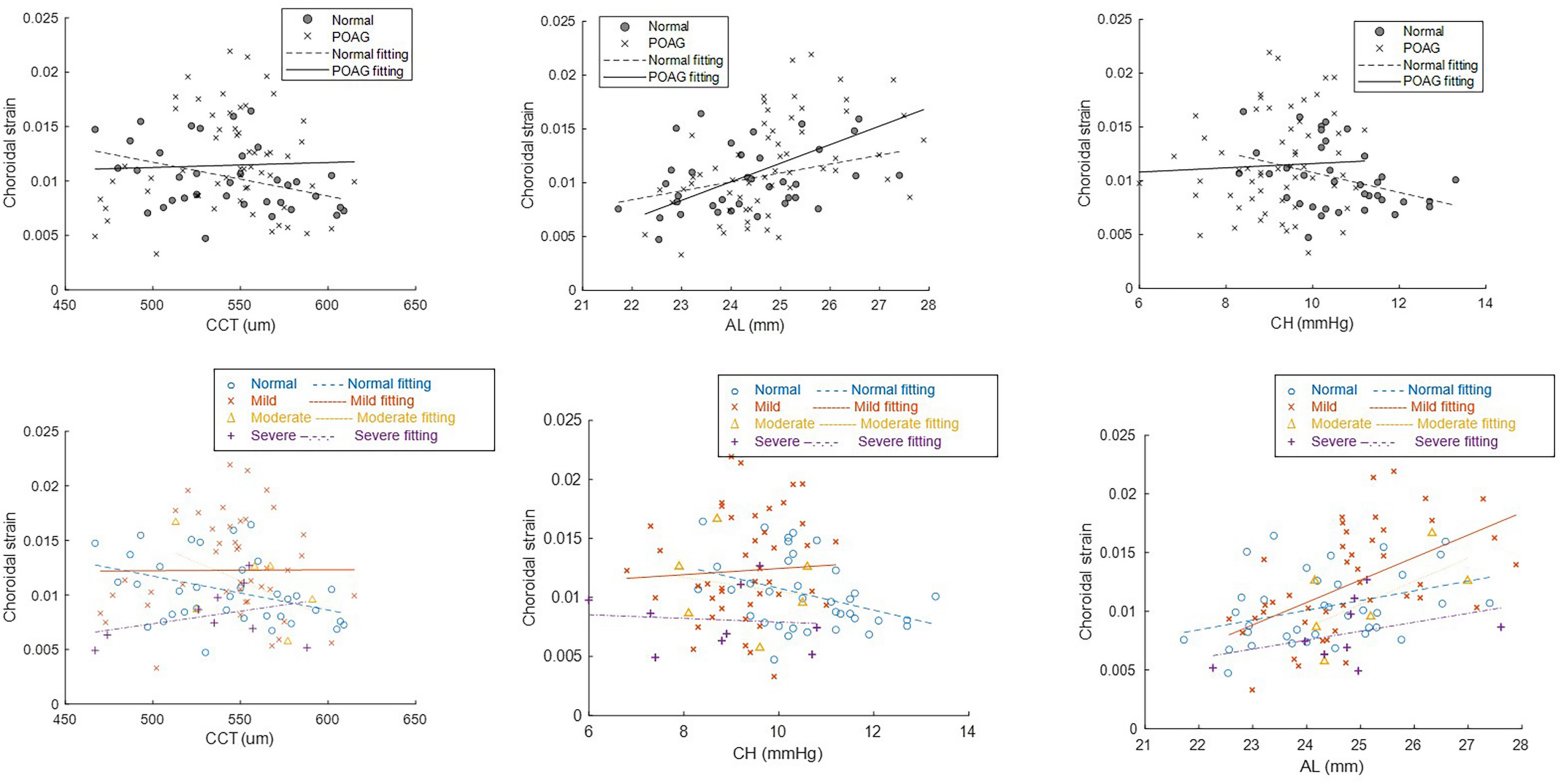
Linear mixed effects regression showing the correlation between choroidal max strain and axial length (AL), corneal hysteresis (CH) and central corneal thickness (CCT) in normal (*N* = 36) and primary open angle glaucoma (POAG) (*N* = 58) eyes and subgroups of mild, moderate and severe POAG.

Choroidal maximal strain exhibited a significant positive correlation with AL in normal eyes (*p* = 0.034, power = 78.3%, β = 8.3e-4, CI = [7e-5, 1.6e-3]), all POAG eyes (*p* = 0.0002, β = 1.7e-3, CI = [8.6e-4, 2.6e-3]), and in of mild POAG subgroup (*p* < 0.001, power = 99.2% β = 0.0019, CI = [0.0009, 0.0029]), but not in moderate and severe POAG subgroups (*p* ≥ 0.119). CMS also exhibited a significant negative correlation with CCT (*p* = 0.026, power = 82.4%; β = −3.1e-5, CI = [−5.8e-5, −4.0e-6]) and CH (*p* = 0.042, power = 74.1%, β = −9.3e-4, CI = [−1.8e-3, −3.6e-5]) in normal eyes, but this was not seen in all POAG eyes nor in subgroups of mild, moderate or severe POAG eyes (*p* ≥ 0.193) (Figure 3).

Choroidal maximal strain also correlated with age in POAG (*p* = 0.049) but not in normal eyes (*p* = 0.153) (data not shown); additionally, CMS did not correlate with IOP, BPA nor MOPP in either group (*p* ≥ 0.076). Because CMS was correlated with AL, we also examined the relationship between AL and CCT and CH but found no significant correlations for either group (AL and CCT: *p* = 0.717 for normal and *p* = 0.328 for POAG; AL and CH: *p* = 0.973 for normal and *p* = 0.066 for POAG).

We performed a sub-analysis to examine whether CMS differed by history of glaucoma surgery or prostaglandin analogue use. No significant difference was detected between POAG eyes with history of glaucoma surgery (*N* = 12) and POAG eyes without surgery (*N* = 46), as well as POAG eyes using prostaglandin analogues (*N* = 43) and POAG eyes not using prostaglandin analogues (*N* = 15) (*p* ≥ 0.35).

## Discussion

Several studies have confirmed the notably strong connection between ocular biomechanics and glaucoma, with thin CCT, lower CH, and longer AL all being identified as risk factors for glaucoma (3–8). Our study provides data that implicates reduced ocular rigidity as an ocular characteristic associated with these findings and may provide an unifying explanation for them.

The reduced scleral rigidity observed in axial myopia is thought to be a result of thinner collagen bundles and reduced volume of the individual collagen fibers (18), and may reflect underlying biomechanical vulnerabilities in the peripapillary sclera and LC. We recently demonstrated an increase in CMS (corresponding to a reduction in ocular rigidity), measured using a customized B-Scan scanning protocol centered at the fovea using FP-OCE, is associated with increasing refractive myopic severity (12). Our current study, by including data on AL, adds data on axial myopia and reveals that decreased ocular rigidity significantly correlates with longer AL in normal and glaucomatous eyes. This is in agreement with earlier studies that used an invasive approach to measure sclera rigidity (9, 19). Wang et al. (20) also found that increased AL was associated with decreased ocular rigidity, calculated as ratio between ocular pulse amplitude using dynamic contour tonometry and pulsatile choroidal blood flow using laser Doppler flowmetry, in POAG, ocular hypertension, glaucoma suspects and normal eyes; our findings are in agreement, using FP-OCE. Sayah et al. (21) also recently reported AL was inversely correlated with ocular rigidity as measured with a non-invasive method using dynamic contour tonometry and SD-OCT; however, they found no correlation between choroidal volume change and AL (as well as with age, blood pressure and IOP) in their non-invasive investigation of pulsatile choroidal volume change using dynamic submacular OCT imaging, and speculated this could be because the choroid tends to be thinner in high myopia.

A thin cornea is considered an independent risk factor for glaucoma (3), but the reason why CCT is connected to glaucoma risk remains unclear. Suggested explanations for the relationship between CCT and glaucoma include the effect of CCT on IOP measurement by applanation, with a thin cornea resulting in a falsely low IOP measurement; and that CCT could be an additional structural risk factor, potentially due to an association between a thin cornea and structural LC characteristics. Some authors have evaluated the relationship between CCT and anterior scleral thickness (22–24), with no relationship being found in POAG but correlation seen in normal tension glaucoma; however the contribution of anterior scleral thickness to glaucoma pathophysiology remains unclear, because scleral thickness varies from anterior to posterior (25). Other authors have evaluated the relationship between CCT and different metrics related to LC and peripapillary sclera thickness, with some demonstrating that CCT was significantly associated with central LC thickness in healthy human eyes (26).

Another corneal biomechanical property implicated in glaucoma is CH, which refers to the ability of the cornea to dampen pressure changes rather than having characteristics of floppiness or rigidity. A lower CH has been found to be a strong predictor of higher glaucoma risk and progression (5, 6). Some authors have shown that in eyes with glaucoma, but not in normal eyes, a lower CH was associated with increased deformation of the optic nerve surface during transient IOP elevation (27); others reported a correlation between a lower CH and a thinner posterior scleral thickness in NTG patients with high myopia (28); and while some authors found no correlation between CH, as measured by ORA, and LC thickness in normal subjects (29), others found a correlation between a lower corneal deformation amplitude, as measured by Corvis Scheimpflug Technology, and a shallower LC depth in POAG (30).

Our study found that ocular rigidity has a positive correlation with both CCT and CH in normal eyes (increased ocular rigidity, measured as decreased CMS, with increasing CCT and higher CH). However, this relationship was not seen in POAG eyes, suggesting that relatively low scleral rigidity may be an associated ocular characteristic for POAG in patients with higher CCT and CH. Pan et al. (31) used a high-frequency ultrasound elastography technique to image the mechanical responses of the cornea and the optic nerve head (ONH) and peripapillary sclera (PPS) to elevated IOP and showed that corneal shear strain was significantly correlated with ONH/PPS shear strain, however they only included normal donor eyes with no known ocular diseases. The question of whether the biomechanical changes are a consequence or part of the pathogenesis of the disease is less clear; some authors have proposed that the chronic mechanical stress on the ONH from glaucoma itself might modify the biomechanical properties of the tissues that support the ONH, leading to remodeling of the extracellular matrix, altering the stiffness, elasticity, and strain response of these tissues (32, 33), which could explain the role of these biomechanical properties as predictive measures of glaucoma development and progression. Although in our study we found no significant difference in CMS between stages of glaucoma, our sample size in the moderate and severe groups were particularly small, and it still might be also possible that scleral rigidity changes in the course of the disease. To address the issue of how scleral changes become manifest during the course of the disease, longitudinal studies will be necessary.

Our study also found a negative correlation between ocular rigidity and age, as measured by choroidal max strain, in POAG but not in normal eyes. With aging, the connective tissue typically becomes more rigid due to an increase in the cross-sectional area of the fibrils and an increase in cross-linking (34). A previous study has reported a positive correlation between ocular rigidity, determined by cannulating the anterior chamber in normal eyes undergoing cataract surgery, and age (9), but others found no correlation (35). Because POAG prevalence increases with age, it is possible that a paradoxical decline in ocular rigidity, or lack of a typical age-related increase in rigidity, may play a role in the pathogenesis of glaucoma.

In summary, we found lower ocular rigidity with older age and increased axial length, and the increase in rigidity seen in normal eyes with increased CCT and CH was not seen in POAG eyes.

Our study has several limitations. While biomechanical changes might be one of the drivers of the pathogenesis of glaucoma, we must highlight that there are multiple factors that contribute to these biomechanical changes, and the relative contribution of each factor is potentially patient specific. Most of the POAG eyes had mild glaucoma (74%), and therefore, our cohort likely lacks sufficient variability to support subgroup analysis by disease severity findings. Some of our POAG subjects had undergone incisional glaucoma surgery, and surgical treatment of POAG can result in changes in CH and, potentially, CCT (36). Additionally, prostaglandins (PGAs) are commonly used as first-line therapy for IOP reduction in patients with glaucoma (36), and the chronic use of PGAs has been shown to be associated with a decrease in the collagen type I level (37, 38), which could have altered the relationship between corneal parameters and scleral rigidity in our POAG eyes; though no significant differences were found in CMS between the two groups, nearly 75% of the glaucoma cohort were taking a PGA. Our choroidal pulsation model assumes that the choroidal max strain is negatively correlated with scleral rigidity with the underlying prerequisite that the force causing the choroidal expansion (the ocular perfusion pressure) stays constant each cardiac cycle, however, IOP, MOPP and BPA vary from person to person and at different times of the day. We should note that we did not find differences in those parameters between the two groups.

In conclusion, we investigated a new and non-invasive clinical measurement of ocular rigidity using FP-OCE. While no significant difference in ocular rigidity was detected in treated glaucoma and normal eyes, ocular rigidity exhibited a significant negative correlation with AL in all eyes, and with age in POAG but not normal eyes. In addition, ocular rigidity significantly increased with increasing corneoscleral stiffness parameters (CCT and CH) in normal eyes, but not in POAG eyes. The sum of these findings suggests that ocular rigidity may be involved in the pathogenesis of POAG; however, confirming a causal relationship between ocular rigidity and glaucoma development and progression will require prospective longitudinal studies.

## Data Availability

The raw data supporting the conclusions of this article will be made available by the authors, without undue reservation.
